# How to measure RNA expression in rare senescent cells expressing any specific protein such as p16^Ink4a^

**DOI:** 10.18632/aging.100536

**Published:** 2013-02-25

**Authors:** Jessie C. Jeyapalan, John M. Sedivy

**Affiliations:** Department of Molecular Biology, Cell Biology and Biochemistry, Brown University, Providence, RI 02912, USA

**Keywords:** Cellular senescence, flow cytometry, immunostaining, RNA purification, transcriptome profiling

## Abstract

Here we describe a carefully optimized method for the preparation of high quality RNA by flow sorting of formaldehyde fixed senescent cells immunostained for any intracellular antigen. Replicative cellular senescence is a phenomenon of irreversible growth arrest triggered by the accumulation of a discrete number of cell divisions. The underlying cause of senescence due to replicative exhaustion is telomere shortening. We document here a spontaneous and apparently stochastic process that continuously generates senescent cells in cultures fully immortalized with telomerase. In the course of studying this phenomenon we developed a preparative fluorescence activated flow sorting method based on immunofluorescent staining of intracellular antigens that can also deliver RNA suitable for quantitative analysis of global gene expression. The protocols were developed using normal human diploid fibroblasts (HDF) and up to 5×10^7^ cells could be conveniently processed in a single experiment. The methodology is based on formaldehyde crosslinking of cells, followed by permeabilization, antibody staining, flow sorting, reversal of the crosslinks, and recovery of the RNA. We explored key parameters such as crosslink reversal that affect the fragmentation of RNA. The recovered RNA is of high quality for downstream molecular applications based on short range sequence analysis, such qPCR, hybridization microarrays, and next generation sequencing. The RNA was analyzed by Affymetrix Gene Chip expression profiling and compared to RNA prepared by the direct lysis of cells. The correlation between the data sets was very high, indicating that the procedure does not introduce systematic changes in the mRNA transcriptome. The methods presented in this communication should be of interest to many investigators working in diverse model systems.

## INTRODUCTION

Replicative cellular senescence is a phenomenon of irreversible growth arrest triggered by the accumulation of a discrete number of cell divisions. *In vivo* studies have implicated cellular senescence as an important tumor suppression mechanism in a variety of human and mouse tissues [1,2]. Cellular senescence has also been linked with aging and age related pathology [3]. Telomere shortening was the first described cause of senescence [4], but many other triggers have since been documented, including oncogene activation, a variety of genotoxic insults, and oxidative as well as other yet poorly understood stresses [5,6]. One central mechanism is the presence of unrepaired or persistent DNA double-strand breaks (DSB), which arise from telomere dysfunction or other genotoxic insults, and signal through the DNA damage response (DDR) pathway to activate the p53 tumor suppressor, leading to the upregulation of the cyclin-dependent kinase (CDK) inhibitor p21 and cell cycle arrest [7].

The second pathway of considerable importance is governed by the pRb tumor suppressor, which is maintained in its active state by the upregulation of the p16 CDK inhibitor [8,9]. The DDR can signal to p16 through mechanisms such as the activation of the p38 MAPK pathway, but the regulation of p16 is not well understood, and likely involves components that are independent of genotoxic stress [10,11]. For example, while the expression of telomerase elongates telomeres and hence prevents their dysfunction and activation of the p53-p21 pathway, immortalization of some fibroblast strains and most epithelial cell types requires the additional silencing of p16 [12-14].

We previously documented that when normal human diploid fibroblasts (HDF) approaching replicative senescence were monitored at the single cell level by immunofluorescence microscopy, p21 and p16 were initially upregulated in different cells [15]. While this suggested the possibility that p21 and p16 were upregulated in response to different triggers, fully senescent cells expressed high levels of both p21 and p16, and expression of hTERT in presenescent cells was sufficient to generate immortalized clones. We report here the unexpected finding that HDF cultures fully immortalized with hTERT continue to generate senescent, p16-positive cells at an appreciable frequency, with no evidence of DDR.

These observations indicate that presenescent and senescent cultures are heterogeneous mixtures of cells with different characteristics and fates [15-17]. This is certainly expected to be the case *in vivo*, where senescent cells are typically found at low frequencies within tissues [18-20], and underscores the need for single-cell techniques to molecularly analyze these rare pools of cells. While laser capture microdissection has been used with some success, these methods are compromised by the poor quality of the recovered RNA, and in the case of senescence, widely dispersed cells. Flow cytometry has important advantages, including the ability to recover substantial numbers of cells, but has mostly been used with antibodies directed at cell surface antigens. Given that p16 is an intracellular antigen, we have developed and report here a preparative method based on formaldehyde crosslinking, followed by crosslink reversal for the recovery of RNA. We can routinely obtain >10^6^ cells, the recovered RNA is of adequate quality for accurate qPCR, microarray and next generation sequencing, and the method should be adaptable to studying many different cellular processes in addition to senescence.

## RESULTS

### Spontaneous and DDR-independent upregulation of p16 in hTERT-immortalized HDF

**Figure 1:**
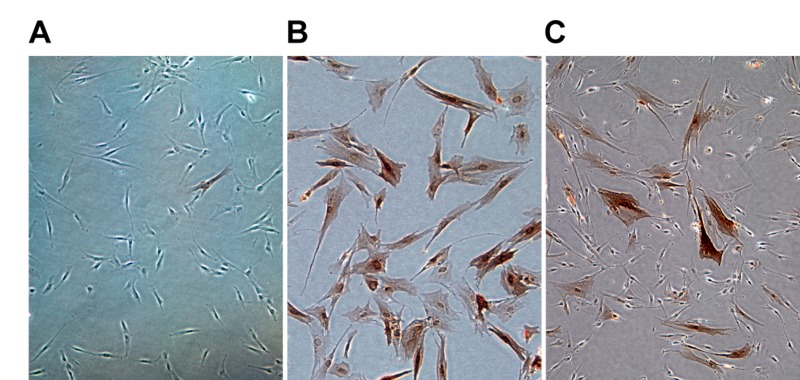
Expression of p16 at the single cell level measured by immunostaining with a p16 antibody followed by immunohistochemical detection **(A)** Non-immortalized HDF (LF1) at early passage. **(B)** LF1 HDF passaged into senescence. **(C)** LF1 cells immortalized with telomerase (LF1/TERT) under conditions of exponential proliferation.

We reported previously that p21 and p16 were upregulated in different cells as cultures of fetal lung HDF approached replicative exhaustion [15]. Cells singly positive for either p21 or p16 were senescent, as verified by staining for the senescence-associated β-galactosidase marker [21] and absence of BrdU incorporation [15]. Ectopic expression of telomerase in early passage cells prevented telomere dysfunction and activation of the p53-p21 pathway [15], and readily yielded immortalized clones, some of which have been extensively propagated [22]. We were thus surprised to find that cultures of telomerase-immortalized HDF (designated LF1/TERT, Methods) contained significant proportions (15-20%) of p16-positive cells (Figure 1). We previously showed that in non-immortalized cultures, p16-positive cells are continuously generated at low levels even at early passage, that this process increases with passage, and is independent of telomere dysfunction and p53-p21 pathway signaling [15]. Apparently, this process continues unabated after immortalization with telomerase.

**Figure 2:**
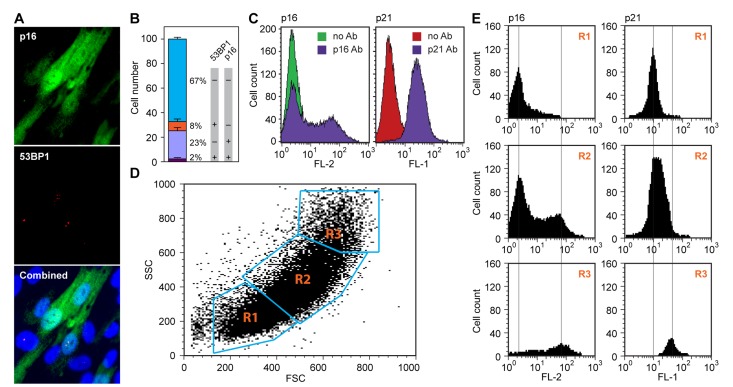
Spontaneous upregulation of the p16-pRb and p53-p21 pathways in hTERT-immortalized HDF **(A)** p16 and 53BP1 proteins were simultaneously visualized in exponentially cycling cultures by *in situ* immunofluorescence staining with antibodies to p16 (top panel, green) and 53BP1 (middle panel, red). Note that p16 shows a diffuse cytoplasmic and a nuclear signal, while 53BP1 stains discrete foci in the nucleus. A merged image including DAPI staining is shown in the bottom panel. **(B)** Quantification of the experiment shown in panel (a). Cells were scored in images of random fields and assigned to one of four categories: double-negative (67%), double-positive (2%), p16 positive only (23%) and 53BP1 positive only (8%). Note the virtual absence of double-positive cells. **(C)** Flow cytometric analysis of cells stained either with a p16 antibody (left panel), or a p21 antibody (right panel). Samples were processed as indicated in Methods. Samples processed without primary antibody were used as negative controls (no Ab). Secondary antibodies were conjugated with Cy3 (p16, left) and Alexa488 (p21, right), and the data were acquired in the FL-2 and FL-1 channels, respectively. **(D, E)** Flow cytometric analysis of cells doubly stained with antibodies to p16 and p21. **(D)** Acquired data were first displayed as a scatter plot of forward scatter (FSC) and side scatter (SSC) parameters, and gates (R1, R2, R3) were drawn around areas of increasing scatter. **(E)** Cells in the gated areas were then analyzed for fluorescence in the FL-1 (p21) and FL-2 (p16) channels, and the data were displayed as histograms. Note that the smallest cells (R1 gate) displayed essentially no p16 staining and low p21 staining, and the largest cells (R3 gate) displayed the highest levels of both p16 and p21 staining.

Further elucidating this telomere-independent, p16-pRb pathway regulated senescence process would be of considerable interest. We first investigated, using single-cell immunofluorescence analysis, the correlation between upregulation of p16 and the DDR (Figure 2 A,B). Using 53BP1 as a sensitive readout of DSB, we found that in exponentially growing LF1/TERT cultures, 53BP1 foci and p16 upregulation occurred mostly in different cells, with only a very small fraction (2%) of double-positive cells. This suggests that p16 is upregulated independently of DDR signaling. The low frequency of DSB (approximately 10% 53BP1-positive cells) is caused by oxidative stress due to atmospheric oxygen, and cannot be eliminated even by culture under physiological (2.5%) oxygen tension (Methods).

The regulation of p16 expression has been of considerable interest. Using immunoblot or qPCR analyses the levels of p16 are seen to rise gradually as cultures approach senescence, while others have argued that p16 is regulated more as a “ON-OFF” switch, with pre-senescent cells containing essentially no p16 [8,9]. Our microscopic data (Figure 1) are more consistent with the switch mechanisms. To investigate this more closely we performed flow cytometry after staining with p16 antibodies, and found a distinct biphasic profile (Figure 2C). Using a monoclonal p16 antibody with essentially no background staining, the lower intensity peak was found to overlap exactly with unstained cells. This clearly biphasic expression pattern is also consistent with the ON-OFF switch mechanisms, with the OFF state being devoid of detectable p16. In contrast, staining with p21 antibody resulted in a largely monophasic peak that was shifted to the right from unstained cells. HDF are known to express low but detectable levels of p21 [15,22], and this pattern is thus consistent with all cells expressing relatively uniform levels of p21 (the 53BP1-positive cells with activated DDR signaling express elevated levels of p21, but because of their low frequency and small degree of p21 induction, are not apparent in this analysis).

A molecular characterization of the p16-positive senescent cells would be of considerable interest, but would have to be performed without interference from the majority of pre-senescent cells as well as the small minority of 53BP1-positive DDR activated cells. It is well known that senescent cells increase in size, and this parameter has been used to flow sort live senescent cells [23]. We therefore stained exponentially growing LF1/TERT cells with antibodies to p16 and p21, gated different regions of a FSC/SSC dot plot, and displayed the FL-1 (p21) and FL-2 (p16) histograms of the selected gates (Figure 2 D,E). The smallest cells (R1 gate, Figure 2E top panels) showed no p16 expression and low p21 expression. The middle gate (R2, Figure 2E middle panels) showed a biphasic p16 profile and slightly elevated p21 expression. The gate with the largest cells (R3, Figure 2E bottom panels) contained relatively few cells, which showed high p16 and p21 levels. Adjusting the gates, or choosing a variety of different gates did not improve the separation. We concluded that this method was unable to provide a sufficient enrichment of p16-positive senescent cells.

### Developing a method for flow sorting based on intracellular antigens

Flow cytometry has been used extensively to purify populations of cells based on immunostaining, but has been mostly limited to cell surface antigens. Immunostaining of intracellular antigens requires fixation to preserve cellular structure and permeabilization to allow antibody access. While these methods have been successfully combined with flow cytometry, the quantitative recovery of good quality RNA has not been adequately documented. Recovery of RNA is however essential to enable high throughput, genome-wide methods of downstream analysis. We therefore systematically approached this limitation.

A commonly used fixative is formaldehyde, which covalently crosslinks proteins and nucleic acids by reacting with primary amino groups. These crosslinks are reversible with heat and salt [24]. The second most common fixation method uses coagulative organic solvents that precipitate proteins and nucleic acids. Return to aqueous conditions can however cause significant losses of the highly soluble nucleic acids [25,26]. Crosslinking with formaldehyde prevents these losses, and is also quite effective in inactivating endogenous RNases. However, while commonly used with DNA, crosslink reversal is problematic with RNA, which, by virtue of its greater chemical reactivity, is significantly fragmented by the heat treatment.

**Figure 3:**
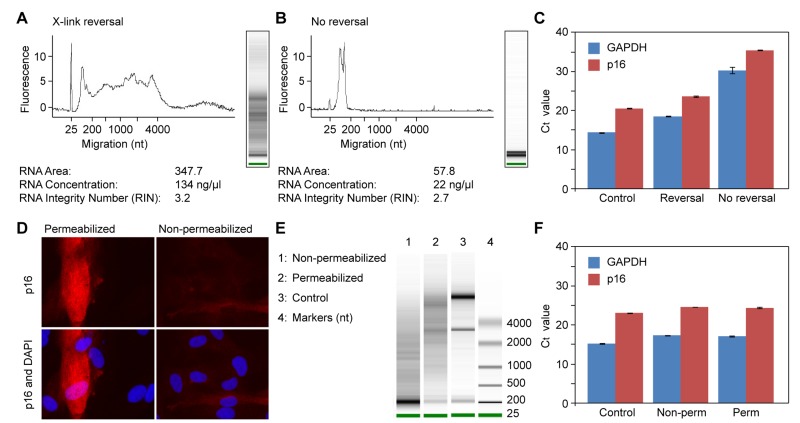
Effects of formaldehyde crosslinking, crosslink reversal, and permeabilization on RNA extraction and quantification of gene expression by qPCR **(A-C)** Effect of crosslink reversal. **(A)** Cells were harvested by trypsinization, fixed with paraformaldehyde in solution, permeabilized with Triton X-100, and crosslinks were reversed by incubation in a buffer containing 200 mM NaCl and 1% SDS for 2 hr at 65°C. RNA was subsequently purified by phenol extraction, ethanol precipitated, and analyzed on a Bioanalyzer instrument. **(B)** Cells were processed as in panel A, but the crosslink reversal step was omitted. **(C)** Equal amounts of RNA preparations (1 μg) from (a) and (b) were reverse transcribed and qPCR was performed with primers to *GAPDH* and p16 (gene symbol *CDKN2A*) genes. Control was total RNA that was prepared directly from cells using Trizol reagent. **(D-F)** Effect of cell premeabilization. **(D)** Cells were grown on cover slips and processed for immunofluorescent staining of p16 using a protocol that includes permeabilization with Triton X-100 (left panels). The permeabilization step was omitted in the right panels. **(E)** Cells were processed as in panel (a), except that for lane 1 the permeabilization step was omitted (cells were incubated for an equivalent amount of time in buffer without Triton X-100). Control was total RNA that was prepared directly from cells using Trizol reagent. **(F)** Equal amounts of RNA preparations (1 μg) from (e), lanes 1-3, were reverse transcribed and qPCR was performed as in panel (c).

We performed pilot experiments to test the effect of crosslink reversal, and found that it was essential (Figure 3A). While some fragmentation was apparent, the ribosomal RNA bands were clearly visible, and the yield in the high mw range was good. Without reversal, the recovery of RNA was very low, and only low mw RNAs were recovered (Figure 3B). Most importantly, qPCR analysis showed that the RNA was of high quality (Figure 3C). While the Ct values were somewhat lower after crosslink reversal, the ratio of p16 to GAPDH was quite constant. Without reversal, the Ct values were very high, but the ratio of p16 to GAPDH was maintained, indicating that even with this poor recovery, the gene expression pattern of p16 was preserved. We also tested the effect of permeabilization. While we found that this was essential for antibody staining (Figure 3D), it did not have a significant affect on the RNA (Figure 3E). In this experiment, the Ct values for p16 and GAPDH were very similar between the control, permeabilized and non-permeabilized samples (Figure 3F). We also tested the effect of trypsin as a method of harvesting the cells, and found that it had no deleterious consequences.

**Figure 4:**
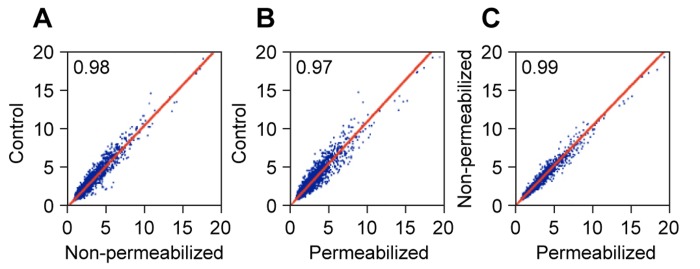
Affymetrix microarray performance of RNA extracted from formaldehyde crosslinked cells RNA was prepared as in Figure 3A and used for expression profiling on Affymetrix Human Gene ST 1.0 arrays. The experiment included both permeabilized and non-permeabilized samples, and RNA that was prepared directly from cells using Trizol reagent as a control. Correlation plots of the expression values (log2 PLIER scores) are shown for 33,297 probesets. Genes with very low expression scores (lowest quartile of all probesets) were excluded from the analysis. **(A)** Control versus Non-permeabilized. **(B)** Control versus Permeabilized. **(C)** Non-permeabilized versus Permeabilized.

As judged by the qPCR analysis the quality of the RNA was high, and the preparations were thus processed for gene expression profiling on Affymetrix Human Gene ST 1.0 arrays (Figure 4). Control RNA was prepared directly from cells without any manipulations by standard methods (Trizol reagent). The genome-wide correlation coefficients between the samples were very high (0.97-0.99). This variability is in the range of stochastic variability between individual arrays hybridized with the same sample.

### Preparative flow sorting of p16-positive senescent cells

**Figure 5:**
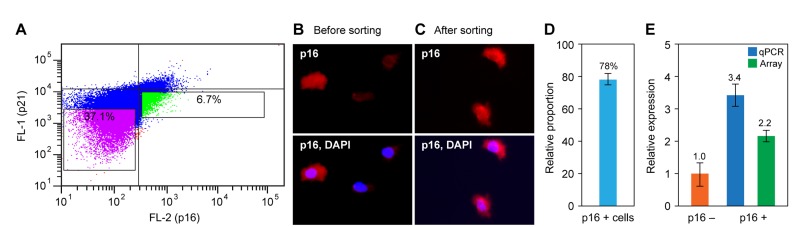
Preparative flow sorting of p16-positive cells and analysis of the recovered RNA **(A)** Cells were doubly stained with p16 and p21 antibodies and processed as described in the Methods. Data from an actual sorting experiment are shown. The gates were set using singly stained and unstained preparations (not shown). The indicated regions were collected: green, p16-positive and p21-negative cells; purple, double negative cells. **(B, C)** After the sort the collected samples were spotted on glass slides and examined by fluorescence microscopy to determine the degree of enrichment. Representative images of (b) the total population and (c) the green-gated region from panel (a) are shown. **(D)** Quantification of p16-positive cells from panel (c). The unsorted population contained 18% of 16-positive cells (not shown). **(E)** RNA was prepared from flow sorted p16-positive and p16-negative cells (green and purple gated regions in panel (a), respectively), and p16 expression was assessed by qPCR and microarray analysis. Data are shown as fold change relative to p16-negative cells, and were normalized to GAPDH expression.

Based on the pilot experiments presented above we developed a scaled up protocol to process up to 5×10^7^ cells with a yield of approximately 1×10^6^ p16-positive cells (Methods). In addition to the issues discussed, it is of obvious importance to stringently control contamination with RNases. This included the use of RNase inhibitors in the incubation steps, bleach-sterilizing the cell sorter, and operating it with DEPC-treated sheath fluid. We have performed this procedure numerous times, with very consistent results. A representative experiment is shown in Figure 5. Note that cells were stained for both p16 and p21, so that not only p16-positive cells could be separated from p16-negative cells, but p16-positive cells could be separated from the small fraction of p21-positive cells that have activated the DDR. Gating was determined in each experiment using unstained and individually stained control preparations; typically, with conservative gating to achieve good discrimination the yield of p16-positive cells was in the range of 5-8% (Figure 5A).

The quality of the immunostaining as well as the accuracy of the sorting was verified by fluorescence microscopy. Small aliquots of cells from the various fractions were spotted on glass slides, air-dried, counter-stained with DAPI, and mounted for microscopy. Before sorting the total cell preparations showed p16-positive cells in the range of 20% (Figure 5B), as previously observed (Figure 2A,B). Clear enrichment was seen after the sort (Figure 5C), which in the experiment shown was quantified at 78% (Figure 5D).

RNA was recovered from the sorted, p16-positive/p21-negative and control (p16-negative/p21 negative) pools of cells and subjected to qPCR and microarray analysis. In the experiment shown (Figure 5E), p16 mRNA expression was enriched 3.4-fold by qPCR and 2.2-fold by microarray. This difference is typical of the higher dynamic range of qPCR.

## DISCUSSION

The primary objective of this communication is to report the development of a preparative flow sorting method based on immunofluorescent staining of intracellular antigens that can also deliver RNA suitable for quantitative analysis of global gene expression. While these objectives have been achieved individually (FACS based on intracellular antigens, high quality RNA prepared from intact flow sorted cells), to our knowledge they have not been combined in a carefully explored, robust protocol.

The main issues are that the cells have to be permeabilized to expose the intracellular antigens to the antibodies (Figure 3D), which however also allows some of the intracelluar RNA to diffuse out from the cells. Thus, a fixation step becomes necessary. The permeabilization step can also release endogenous RNases, as well as allow extracellular contaminating RNases to access the RNA. Formaldehyde is a very effective fixative that covalently crosslinks proteins and nucleic acids, which essentially eliminates the losses of RNA, and also works well to inactivate endogenous RNases. However, this also creates a problem of releasing the RNA from the crosslinks, as the crosslink reversal requires heat and salt, to which RNA is intrinsically susceptible due to chemical fragmentation.

This creates a compromise situation, where the formaldehyde fixation and crosslink reversal need to be balanced against each other. For example, in the laser capture microdissection methodologies, the extensive formalin fixation of the tissues necessitates aggressive crosslink reversal, which in turn typically results in extensive RNA fragmentation. The protocol we present here works well for cultured HDF, but may need to be further optimized for other cell types, and almost certainly for tissues. Attention to the parameters discussed above should facilitate this optimization. Control of endogenous and exogenous RNases is of obvious importance, and while we have not found it necessary, the RNAlater reagent (Qiagen) has been found to be compatible with antibody staining [27], and could be used in the initial steps of our protocol.

Heat-induced fragmentation of RNA introduces mostly random breaks into the phosphodiester backbone. Relatively mild fragmentation will thus rapidly obscure the rRNA bands when visualized by electrophoresis, and drop the RIN values on the Bioanalyzer instrument (Agilent) into the 3-5 range. Such samples will display as broad smears in the high mw (200-4000 nt) range. However, because of the relatively large size of these fragments, in our hands such preparations perform very well in applications based on short range sequence analysis, such qPCR, hybridization microarrays, or next generation sequencing. More extensive fragmentation, typically due to RNase contamination, results in loss of the high mw migrating material, and accumulation of signal in the low mw (<200 nt) range. Such preparations do not perform well in downstream applications.

We processed RNA samples obtained by our procedure all the way through Affymetrix Gene Chip expression profiling, and compared the gene expression patterns to those obtained using standard RNA preparation (direct lysis of intact cells in Trizol reagent). The very high correlation coefficients between the data sets (Figure 4), which were generated from the same starting material (exponentially growing LF1/TERT cells), indicate that none of the steps in our protocol introduce systematic changes (losses or gains) of individual mRNAs across the whole transcriptome.

The biological observation that stimulated the development of these protocols was the surprising discovery that fully immortalized HDF cultures continue to generate senescent, p16-positive cells, with no evidence of DDR. The p16-positive cells do not cycle and are continuously diluted as the cultures proliferate, while new p16-positive cells are generated in the cultures from proliferating cells, thus maintaining a steady-state frequency of p16-positive cells of approximately 20%. We still do not understand the upstream signaling that triggers these spontaneous and apparently stochastic senescence events, and the ability to obtain these rare cells in amounts sufficient for molecular studies will greatly aid these studies. The methods presented in this communication should be of interest to many investigators working in diverse model systems.

## METHODS

### Cell Culture

The hTERT-immortalized human lung fibroblast cell line LF1/TERT [22] was cultured in Ham's F10 medium supplemented with 15% fetal bovine serum, 1% glutamine and 1% penicillin/strepto- mycin. Incubation was at 37°C in an atmosphere of 93% N_2_, 5% CO_2_ and 2.5% O_2_. Cultures were passaged at 1:4 dilution upon reaching 80-90% confluence.

### In situ Immunofluorescence

Cells were grown on glass cover slips and immunofluorescent staining was performed as described [15]. Primary antibodies were as follows: p16, mouse monoclonal (cell line JC8), Santa Cruz (SC-56330); p21, rabbit polyclonal (c19), Santa Cruz (SC-397); 53BP1, rabbit polyclonal, Novus Biologicals (NB100-304). Secondary antibodies conjugated with Alexa488 and Cy3 were from Invitrogen. During flow cytometry experiments (see protocols below), small aliquots of cells were removed from the final preparations, air dried on a microscope slide, mounted with DAPI-containing mounting medium, and visualized in a fluorescence microscope to ascertain the quality of the staining. Images were acquired on a Zeiss Axiovert 200M fluorescence microscope, housed in the Brown BioMed Leduc Bioimaging Facility (http://www.brown.edu/Facilities/ Leduc_Bioimaging_Facility).

### Extraction of Total RNA from Unfixed Cells

Culture plates were rinsed with ice-cold PBS and Trizol reagent (Invitrogen) was added (1 ml per 10 cm dish). After incubation for 3 min at room temperature cells were scraped into the reagent with a Teflon cell harvester. Further processing was according to the manufacturer's instructions. The isopropanol precipitated RNA pellet was washed with 70% ethanol, air dried, resuspended in 0.1 × TE (1 mM Tris-HCl pH 8.0, 0.1 mM EDTA) and treated with RNase-free DNase (RQ1, Promega, M610A) for 30 min at 37°C (1 unit DNase per 1 μg of RNA). DNase was inactivated with the stop solution provided and incubation at 65°C for 10 min. RNA yield was quantified using a NanoDrop spectrophotometer (Thermo Scientific), and RNA quality was assessed using a Bioanalyzer instrument (Agilent Technologies).

### Real Time Quantitative PCR

RNA was converted to cDNA using random hexamers and the TaqMan Reverse Transcriptase Kit (Applied Biosystems). qPCR was performed using the SYBR Green Master Mix in a 7500 Fast Real-Time PCR System (Applied Biosystems). Standard PCR conditions recommended by the manufacturer were used: denaturation at 95°C for 15 sec, annealing and extension combined at 60°C for 1 min, total of 40 cycles. Primer sequences were as follows: GAPDH, forward ggagtcaacggatttGG tcgt, reverse gttgaggtcsstgaaggggtca; p16, forward cggaaggtccctcagacatc, reverse ccctgtaggaccttcggtga.

### Microarray Analysis of Gene Expression

All reagents and instruments were from Affymetrix. 100 ng of total RNA was amplified with the 3' Express Kit. Fragmented and labeled cRNA was hybridized to Human Gene ST 1.0 arrays overnight at 45°C. Arrays was stained using the Hybridization-Wash-Stain kit and Fluidics Script FS40 0002 on the 450 fluidics station. The arrays were scanned using the 3000 G7 scanner. These procedures were performed by the Brown BioMed Genomics Core Facility (http://www.brown.edu/ Research/CGP/core/equipment). The Affymetrix Power Tools software package was used to normalize the arrays, and the Probe Logarithmic Intensity Error (PLIER) algorithm was used to generate expression values for all probe sets. Correlation plots comparing PLIER scores for all probe sets between different conditions (Figure 4) were generated using R software.

### Flow Cytometry

All analytical procedures (Figure 3C-E) were performed on a FACSCalibur instrument (Becton Dickinson). Data on a minimum of 10,000 events were collected for each condition. Preparative sorting was performed by the Brown BioMed Flow Cytometry and Sorting Facility (http://biomed.brown.edu/mmi/flow_facility) on a FACSAria Cell Sorter (Becton Dickinson), using a 488 nm laser for excitation in the FL-1 (FITC) and FL-2 (PE) channels. Instrument stability and alignment were monitored by running flow check beads. Since fibroblasts are large and irregularly-shaped cells, a 100 μm nozzle was used and run at low pressure (20 psi), for sort speeds in the range of 2000-3000 cells/sec. A typical sorting run took 4-5 hr to complete.

### Antibody Staining of Cells in Suspension

The basic analytical scale protocol started with ~1.5 × 10^6^ cells (one 10 cm dish at 70-80% confluence), which were detached with 1 ml Trypsin-EDTA (0.25%, Invitrogen, 15050) at room temperature. All subsequent steps were performed with solutions chilled to 0°C, unless indicated otherwise. The cell suspension was combined with 9 ml of ice cold PBS, mixed well by inverting the tube, and centrifuged at 1000 × g for 5 min at 4°C (recovery of cells from suspension was always accomplished by centrifugation under these conditions). At all stages pellets were gently but thoroughly resuspended by repeated pipetting to obtain single-cell suspensions. The PBS wash was repeated once, and the cell pellet was resuspended in 2 ml of PBS. The cell suspension, held in a 15 ml polypropylene centrifuge tube (Corning, 430790) was placed in a vortexer, and while being continuously mixed at low speed, an equal volume of 8% paraformaldehyde (in PBS) was added dropwise (total time of addition was approximately 60 sec). These procedures were necessary to avoid the clumping of cells into doublets and larger aggregates. The tube was placed in a rotating wheel and inverted at 10 rpm for 15 min at 4°C. Cells were recovered by centrifugation, the supernatant was removed, and the cells were resuspended in 10 ml of PBS containing 0.25 M glycine. The total time from the beginning of paraformaldehyde addition to the quenching of the crosslinking with glycine was 20 min. The cells were washed once with 10 ml of PBS, and the cell pellet was resuspended in 10 ml of PBS containing 0.2% Triton X-100. The permeabilization was allowed to proceed for 20 min at 4°C (this and all subsequent incubations were without agitation), after which the cells were recovered by centrifugation. Cells were resuspended in 1 ml of blocking solution containing 3% bovine serum albumin (Fraction-V, Fisher, BP1600-100) in PBS, and incubated for 1 hr at 20°C. Cells were recovered by centrifugation, resuspended in 1 ml of primary antibody solution (1:100 dilution of antibody in PBS with 3% BSA), and incubated for 2 hr at 20°C. Cells were recovered by centrifugation, washed three times with 10 ml of PBS, resuspended in 1 ml of the secondary antibody solution (1:1000 dilution of secondary antibody in PBS with 3% BSA) and incubated for 1 hr at 20°C. Incubation with the secondary antibody was in a foil-covered tube, and all subsequent steps were performed under dimmed light conditions. Finally, cells were recovered by centrifugation, washed three times with PBS, and resuspended in 1 ml of PBS. This protocol was used for all analytical scale flow cytometry, such as shown in Figure 2. Throughout this procedure it is important to perform all centrifugations in a swinging bucket rotor, to avoid loss of cells due to sticking to the sides of the centrifuge tube.

### Antibody Staining for Cell Sorting and RNA Recovery

To achieve these goals two criteria had to be met: 1) the basic protocol (above) had to be significantly scaled up, and 2) RNA degradation had to be stringently avoided and controlled. For 1), cells were grown in 15 cm dishes (~3 × 10^6^ cells/plate), and multiple plates [15-20] were harvested for one experiment. Up to 3 × 10^7^ cells could be processed by the basic protocol (above, in one 15 ml centrifuge tube) without changing the volumes or compromising the quality of the antibody staining. A typical experiment started with 4-5 × 10^7^ harvested cells, which were processed for antibody staining in parallel in two 15 ml tubes. As the yield from the antibody staining was in the range of 60%, the flow sorting was started with 2.5-3.0 × 10^7^ stained cells. Gating was set to obtain 80% (or greater) enrichment for p16-positive cells, which resulted in ~5% of total cells being selected, for a total of 1-1.5 × 10^6^ cells in the final sorted fraction. For 2), formaldehyde treatment is an effective way to inactivate endogenous RNases present in the cells. To minimize any residual activity, RNase inhibitor (RNasin Plus, Promega, N2611) was added to all incubation steps (blocking, primary and secondary antibody staining) at 1000 units/ml, as well as during storage in PBS prior to sorting. To eliminate contamination with exogenous RNases, water and solutions such as PBS and sheath fluid, were treated with diethylpyrocarbonate (DEPC; 0.1% followed by autoclaving). Where possible RNase-free reagents were purchased and made up with DEPC-treated water. Sterile DEPC-treated glassware or single-use plastic-ware were used throughout. The cell sorter was rinsed extensively with 0.1% bleach, followed by DEPC-treated water, and finally operated with DEPC-treated sheath fluid.

### Extraction of RNA from Sorted Cells

During the sorting cells were collected into 15 ml centrifuge tubes, which were kept on ice during the sort. After sorting, cells were recovered by centrifugation at 2200 × g for 15 min at 4°C and resuspended in a small volume of PBS. A small aliquot of cells was removed, mounted on a microscope slide (see above), and examined by fluorescence microscopy to determine the enrichment of p16-positve cells that was achieved. To reverse the formaldehyde crosslinking, cells were incubated in 400 μl of 200 mM NaCl, 10 mM Tris-HCl pH 8.0, 1 mM EDTA and 1% SDS, for 2 hr at 65°C. An equal volume of phenol-chloroform was added, the sample was vortexed well to emulsify, and centrifuged at top speed in a microfuge for 5 min at 4°C. The aqueous phase was removed to a new tube. An additional volume of 1 × TE (10 mM Tris-HCl pH 8.0, 1 mM EDTA) buffer was added to the organic phase and re-extracted. The second aqueous phase was combined with the first one, and the pooled fractions were extracted again with an equal volume of fresh phenol-chloroform. The aqueous phase was removed, made up to 0.3 M and 20 μg/ml of sodium acetate and glycogen, respectively, and 2 volumes of 95% ethanol were added. The sample was incubated at −20°C overnight (or −80°C for 30 min), and the precipitate was collected by centrifugation in a microfuge at top speed for 10 min at 4°C. The pellet was washed with 70% ethanol, air dried, and dissolved in 0.1 × TE buffer. Samples were digested with RNase-free DNase (as above), the DNase was inactivated, the RNA was collected by precipitation with two volumes of ethanol, washed with 70% ethanol, and dissolved in 20 μl of 0.1 × TE buffer. The yield was up to 200 ng/μl, typically 2-3 μg of total RNA per experiment.
